# Novel interactions of CLN5 support molecular networking between Neuronal Ceroid Lipofuscinosis proteins

**DOI:** 10.1186/1471-2121-10-83

**Published:** 2009-11-26

**Authors:** Annina Lyly, Carina von Schantz, Claudia Heine, Mia-Lisa Schmiedt, Tessa Sipilä, Anu Jalanko, Aija Kyttälä

**Affiliations:** 1 National Institute for Health and Welfare (THL), Biomedicum Helsinki, Finland and FIMM, Institute for Molecular Medicine in Finland

## Abstract

**Background:**

Neuronal ceroid lipofuscinoses (NCLs) comprise at least eight genetically characterized neurodegenerative disorders of childhood. Despite of genetic heterogeneity, the high similarity of clinical symptoms and pathology of different NCL disorders suggest cooperation between different NCL proteins and common mechanisms of pathogenesis. Here, we have studied molecular interactions between NCL proteins, concentrating specifically on the interactions of CLN5, the protein underlying the Finnish variant late infantile form of NCL (vLINCL_Fin_).

**Results:**

We found that CLN5 interacts with several other NCL proteins namely, CLN1/PPT1, CLN2/TPP1, CLN3, CLN6 and CLN8. Furthermore, analysis of the intracellular targeting of CLN5 together with the interacting NCL proteins revealed that over-expression of PPT1 can facilitate the lysosomal transport of mutated CLN5_FinMajor_, normally residing in the ER and in the Golgi complex. The significance of the novel interaction between CLN5 and PPT1 was further supported by the finding that CLN5 was also able to bind the F_1_-ATPase, earlier shown to interact with PPT1.

**Conclusion:**

We have described novel interactions between CLN5 and several NCL proteins, suggesting a modifying role for these proteins in the pathogenesis of individual NCL disorders. Among these novel interactions, binding of CLN5 to CLN1/PPT1 is suggested to be the most significant one, since over-expression of PPT1 was shown to influence on the intracellular trafficking of mutated CLN5, and they were shown to share a binding partner outside the NCL protein spectrum.

## Background

Neuronal ceroid lipofuscinoses (NCLs) are the most common group of children's progressive neurodegenerative disorders with an estimated incidence of 1:12500 in the USA and Nordic countries and approximately 1:100 000 worldwide [reviewed in [1,2]]. NCL disorders are mostly recessively inherited, and to date, eight different genes have been characterized to underlie these diseases [3-5]. Despite having genetic heterogeneity, NCL diseases resemble each other both clinically and neuropathologically. The clinical course varies from severe congenital disease to milder adult-onset forms. NCLs are phenotypically expressed by progressive mental deterioration, blindness, epileptic seizures and premature death. The pathological findings of these lysosomal storage disorders include intracellular accumulation of autofluorescent lipopigment with variable ultrastructural appearance as well as progressive loss of neocortical neurons. The major component of the intracellular storage material is either the subunit c of the mitochondrial ATP synthase [6] or sphingolipid activator proteins A and D [5,7]. More recent analyses of different mouse models of NCL have exposed additional common features in the brain pathology [reviewed in [8]].

The proteins encoded by NCL genes are localized in different compartments of the secretory pathway. Palmitoyl protein thioesterase 1 (PPT1; CLN1), tripeptidyl-peptidase 1 (TPP1; CLN2) and cathepsin D (CLN10) are soluble lysosomal enzymes [9-11]. CLN3 and the recently identified MFSD8 (CLN7) are transmembrane proteins localizing mainly to the late endosomal/lysosomal compartments [4,12]. Two of the NCL proteins, CLN6 and CLN8, reside mainly in the ER [13,14]. Despite the fact that many NCL proteins were characterized already a decade ago, the physiological functions of most NCL proteins are still not known and neither are the molecular connections between them understood [15]. CLN5 is a glycoprotein disrupted in the Finnish variant late infantile form of NCL (vLINCL_Fin_) [16,17]. The subcellular localization of overexpressed CLN5 has been studied in BHK-21, HeLa and COS-1 and cells, where it was found to be lysosomal [16,18]. In neuronal cells, CLN5 is also present in the cellular extensions, but the specific organelle localization in neurons is still unidentified [16]. PPT1/CLN1 and CLN3 are also found in neuronal axons, where CLN1 has been localized to synaptic vesicles and CLN3 to synaptosomes [19-21]. Controversial data has been reported about the solubility of the CLN5 protein [16,18,22,23], but both human and mouse CLN5 have been found in the mannose 6-phosphoproteome, supporting the presence of soluble CLN5 variants [24,25]. Overall, the CLN5 protein is not well conserved, lacks protein homologues and is currently poorly characterized. Very little is also known about the interactions between different NCL proteins. Vesa and co-workers have shown by co-immunoprecipitation and in vitro binding assays that CLN5 interacts with both CLN2 and CLN3 [23], but the result has not been verified.

To obtain more knowledge of common pathways in NCLs, we examined the interactions of CLN5 with other NCL proteins utilizing pull-down and co-immunoprecipitation analyses. We report four novel interactions for CLN5 and show that PPT1/CLN1 and CLN5 are connected at the level of intracellular trafficking.

## Methods

### Recombinant CLN5-cDNA constructs

Mouse *Cln5*-cDNA lacking the suggested signal sequence (aa 26-341) was cloned in frame with an N-terminal GST-tag in a pGEX2T vector (Amersham Biosciences). For the co-immunoprecipitation assay, the full-length *mCln5*-cDNA (aa 1-341) was cloned into the pcDNA3.1A/Myc-His expression vector (Invitrogen) to produce a CLN5 protein with a C-terminal tag. The trafficking-deficient human CLN5-TD (CLN5 flag330) protein was obtained by inserting an intramolecular flag sequence after amino acid 330 into the full-length CLN5 (aa 1-407) pCMV5 construct, using the QuickChange Site-Directed mutagenesis kit. All constructs were verified by sequencing.

### Cell culture, transfection and immunofluorescence analyses

HeLa and COS-1 cells were cultured in Dulbecco's modified Eagle's medium (DMEM), supplemented with 10% fetal calf serum (FCS) and 1% antibiotics (penicillin/streptomycin). For transient transfections, cells were plated on 6-well plates and the transfection was performed with Lipofectamine™ 2000 (Gibco Life Technologies) or Fugene HD (Roche Diagnostics) transfection reagents according to manufacturers' instructions. For immunofluorescence analysis, transiently transfected cells were grown on coverslips, fixed with ice-cold methanol 48 h after transfection and stained with the specific antibodies described below. The labeled coverslips were mounted using GelMount (Biomeda Corp., Foster City, CA) and visualized using Leica DMR confocal microscope with TCS NT software (Leica Microscope and Scientific Instruments Group). Adobe Photoshop and Adobe Illustrator softwares were used for image processing. All transfection and immunofluorescence experiments were repeated at least three times.

### Antibodies

The human CLN5 protein was detected either with a polyclonal rabbit antibody (1RmI-4) raised against GST-mCLN5 (aa 40-284) or with a guinea pig antibody (1GmII-3) raised against GST-mCLN5 (aa 40-341) [16]. PPT1 and CLN2 were detected with anti-PPT1 antibodies (WB: rabbit polyclonal antibody 8414, 1:500 [19]; IF: rabbit polyclonal antibody AA-PPT1, 1:200 [26] and mouse anti-CLN2 antibody 8C4, 1:20 [27] kindly provided by Drs. Kida and Golabek (New York, USA). CLN3 was detected with the peptide antibody 385 [21]. CLN8 was detected with a rabbit peptide antibody 391 (1:200) [13] and mouse monoclonal anti-HA antibody (Boehringer Mannheim). The mouse monoclonal anti-LAMP-1 antibody H4A3 (WB, IF 1:200) was obtained from the Developmental Studies Hybridoma Bank (University of Iowa, Iowa City). The mouse monoclonal anti-FLAG antibody was from Sigma and the mouse anti c-myc antibody (9E10) and the anti-his antibody from Santa Cruz Biotechnology. The mouse monoclonal PDI (Protein Disulfide Isomerase) antibody was obtained from Stressgen. The α- and β-subunits of the mitochondrial ATP synthase were detected with monoclonal antibodies from Molecular Probes. The secondary antibodies used for the immunofluorescence analyses were from Jackson ImmunoResearch and HRP-conjugated antibodies used for Western blot detection were from DAKO.

### Mice

*Cln1*^-/- ^(*Ppt1*^Δex4^) mice [28] and *Cln5*^-/- ^mice [29] used in this study were maintained on a congenic C57/BL6J strain background. The study has been carried out following good practice in laboratory animal handling and the regulations for handling genetically modified organisms, and it was approved by the Laboratory Animal Care and Use Committee of the National Public Health Institute, Helsinki. Tissues for liver extraction were from adult mice.

### Quantitative Real-time PCR

Real-time PCR was performed as described in [29]. In short, *Cln5*^-/-^, *Cln1*^-/- ^and wild-type cortices were prepared and total RNA was extracted using RNeasy Mini kit (Qiagen) according to the manufacturer's instructions, followed by quantification by spectrophotometry. To average out the inter-individual variability, RNA was extracted from the whole cortical area of four *Cln5*^-/- ^mice, four *Cln1*^-/- ^mice and four of their respective wt littermates and pooled together into two wild-type and two knock-out samples. To eliminate genomic DNA, RNA was treated with DNAse I (Roche, Mannheim, Germany). The RT reactions were carried out on 300 ng of RNA using TaqMan Reverse transcription kit with Random hexamer primers (Applied Biosystems, Foster City, CA) as recommended by the manufacturer. TaqMan Gene Expression Assays of selected genes were purchased from Applied Bio systems (CLN5 Mm00515002_m1, PPT1 Mm00727515_s1). The mRNA expression levels of these genes and a standard house-keeping gene, mouse TATA-box binding protein (Tbp, Mm 00446973_m1), were quantified using real-time PCR analysis (TaqMan chemistry) on an ABI prism 7700 sequence detection system (PE Applied Bioscience, Warrington, UK). The PCR reactions (25 μl) were carried out in triplicate with TaqMan Universal Master Mix according to the manufacturer's instructions using the following parameters: 50°C for 2 min, 95°C for 10 min, 50 cycles of 95°C for 15 sec, 60°C for 60 min. Relative levels of the selected genes were calculated using the ΔΔC_T _method [30] as described previously [29]. The absolute change in expression level is given by 2^-ΔΔCT^. For illustrative purposes, the value for the wt gene expression was set to 1 and CLN5 as well as PPT1 expression values are shown relative to that.

### GST pull-down assays

The GST-mCLN5 fusion construct was expressed in *E. coli *and purified by binding to the glutathione-Sepharose 4B beads (Amersham Biosciences) for 2 h at 4°C. For the search of NCL binding partners, cytosolic extracts of COS-1 cells were prepared by lysing the cells in lysis buffer (25 mM HEPES pH 7.4, 150 mM NaCl, 1 mM EDTA, 0.5% Triton X-100, 0.5 mM MgCl_2 _and protease inhibitors) and removing the cell debris by centrifugation. GST vector control and GST-mCLN5 fusion protein were incubated with 4 ml of COS lysates (~2 mg/ml) overnight at 4°C. The beads were washed five times with lysis buffer and the samples were separated by SDS-PAGE under reducing conditions, immunoblotted and probed for different NCL proteins.

The α- and β-subunits of the mitochondrial ATP synthase were pulled down with GST-mCLN5 and GST-hPPT1_28-306 _[31] from the enriched lysosomal/mitochondrial fraction originating from the mouse liver. Briefly, mouse liver of wt, *Cln5*^-/- ^or Cln1^-/- ^mouse was homogenized (1 g of liver in 5 ml of HB) in HB buffer (320 mM saccharose, 4 mM HEPES pH 7.4, 1 mM MgCl_2_, 0.5 mM CaCl_2 _+ protease inhibitors) and centrifuged at 3500 rpm for 10 min at 4°C. The post-nuclear supernatant was transferred to clean tubes and centrifuged again at 14 000 rpm for 15 min at 4°C. The pellet was then dissolved with 1.5 ml of EB buffer (100 mM NaCl, 50 mM HEPES pH 7.4, 5 mM MgCl_2_, 0.5% Triton X-100 + protease inhibitors). The GST pull-downs were done by incubating 1 ml (2.5 mg/ml) of enriched liver lysosomal/mitochondrial fractions with different GST fusion proteins (GST, GST-mCLN5, GST-hPPT1) over night at 4°C. The beads were then carefully washed with the EB buffer. Samples were separated by SDS-PAGE, immunoblotted and probed for α- and β-subunits. All pull-down experiments were repeated at least twice.

### Co-immunoprecipitation assay

PPT1/CLN1 was transiently expressed together with mCLN5-myc/his or with an OPR1L-his [32] control protein in COS-1 cells. The cells were lysed into an immunoprecipitation buffer (IP buffer: 10 mM Hepes pH 7.4, 150 mM NaCl, 0.5 mM MgCl_2_, 10% glycerol, 0.5% Triton X-100, + protein inhibitor cocktail) and the cell debris was removed by centrifugation. Immunoprecipitation was done by adding a his-specific antibody (1 μg/ml) and incubating the samples on ice for three hours. Immunocomplexes were then captured by incubation with Protein A/G Plus agarose beads (Santa Cruz) over night at 4°C. Agarose beads were then gently centrifuged (3000 rpm for 30 sec.) and washed three times with the IP-buffer. Samples were separated by SDS-PAGE, immunoblotted and probed for PPT1/CLN1.

## Results

### Interactions of CLN5 with other NCL proteins

**Figure 1:**
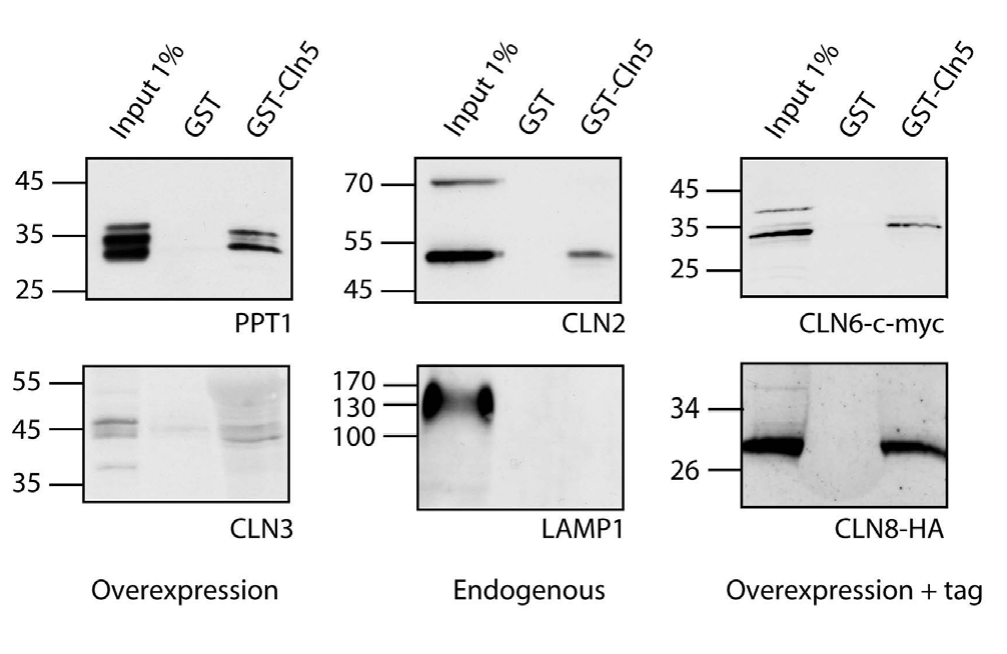
**Interactions of CLN5 with other NCL proteins**. The mouse *Cln5*-cDNA (coding for aa 26-341) was expressed as GST fusion protein and used to pull down NCL proteins from COS-1 cell lysates. PPT1, CLN3, CLN6-c-myc and CLN8-HA were transiently overexpressed in COS-1 cells and pulled down with GST-mCLN5 and GST vector control. CLN2 and LAMP-1 proteins were endogenous. The bound proteins were immunoblotted and detected with specific antibodies listed in Methods section.

The possible interactions of CLN5 with other NCL proteins were investigated with GST pull-down experiments. A GST-CLN5 fusion protein expressing the mouse CLN5 lacking the signal sequence (GST-mCLN5^aa26-341^) was used to pull down endogenous NCL proteins from both mouse brain extract and HeLa cell lysates (Additional file 1). To avoid the possible false negative binding results due to low endogenous levels of most NCL proteins, the pull-downs were also prepared from lysates of COS-1 cells transiently transfected with different NCL proteins (Fig. 1). The bound proteins were identified by Western blot analyses. Vesa and co-workers have previously shown by co-immunoprecipitation and in vitro-binding assays that CLN5 interacts with CLN2 and CLN3 [23]. In our pull-down experiments, we could also capture CLN2 and CLN3 with GST-mCLN5 (Fig. 1), which was considered to serve as a positive control for our further pull-down analyses. The CLN2 antibody recognized both the 68 kDa precursor and the mature, lysosomal 48 kDa form of CLN2 [33] in the cell lysate, but only the mature form showed prominent binding with GST-mCLN5. This suggests that the interaction between CLN5 and CLN2 most likely occurs in the late endocytic compartments. Three novel interactions between NCL proteins were additionally discovered, as PPT1/CLN1, CLN6 and CLN8 were also shown to bind to GST-mCLN5 (Fig. 1). All proteins except CLN8 could also be pulled down endogenously from brain and HeLa cell lysates (Additional file 1). The specificity of the pull-down experiments was tested by using LAMP-1, a lysosomal membrane protein, as a control. It showed no binding to GST-mCLN5 (Fig. 1). After LAMP-1 detection, the membrane was reprobed with anti-CLN2 antibody as a positive control for the pull-down experiment (data not shown). Altogether, the GST-mCLN5 interactions found here cover both soluble and transmembrane NCL proteins residing both in the ER and the lysosomes, thus suggesting that CLN5 has the capacity to bind different NCL proteins at different cellular localizations. This observation indicates that CLN5 may play a role as a factor that connects different NCL proteins.

### Analyses of intracellular trafficking of CLN5 together with the interacting proteins

To dissect the role of CLN5 interactions with other NCL proteins *in vivo*, we first compared the localization of the newly characterized interaction partners in wild type and *Cln5*^-/- ^mouse fibroblasts [29]. Fibroblasts were transiently transfected with constructs producing different NCL proteins (CLN1, 3, 6 and 8, whereas CLN2 could be detected endogenously) and fluorescently labeled with antibodies against the NCL proteins as well as specific organelle markers. However, no differences in the localization of any of the interacting NCL proteins could be detected between the wild type and *Cln5*^-/- ^cells (data not shown). We also studied the possible effects of simultaneous overexpression of wild type hCLN5 and the interaction partners on the protein localization in HeLa cells. Transient overexpression did not influence the localization of CLN1, CLN2, CLN6 or CLN8 (data not shown). The hydrophobic CLN3 was found in lysosomes, but was often detected also in the ER together with CLN5, most probably reflecting a folding defect in a situation in which two proteins are overexpressed (data not shown). This could reflect an overloading of the ER protein folding machinery.

**Figure 2:**
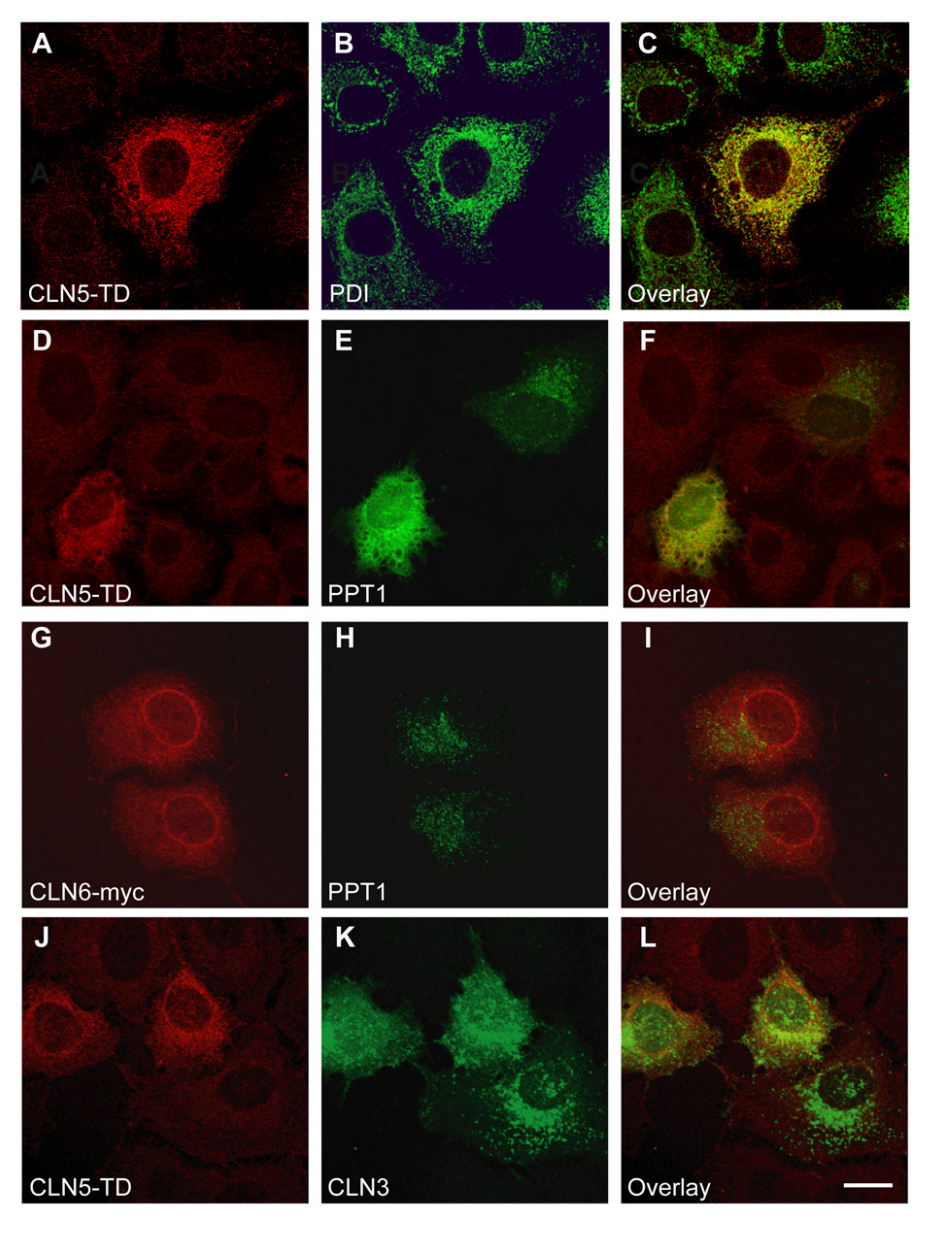
**Consequences of the ER-resident trafficking deficient CLN5 on the localization of PPT1/CLN1 and CLN3 in HeLa cells**. HeLa cells were transiently transfected with the trafficking deficient CLN5 (CLN5-TD) carrying the intramolecular flag-tag after aa 330, alone, or together with wild type PPT1 or CLN3. The cells were fixed with methanol 48 h post transfection, stained and analyzed by confocal microscopy. CLN5-TD co-localized with the ER marker PDI (**A-C**). When transfected together with PPT1, CLN5-TD retained the wild type PPT1 also in the ER (**D-F**), (compare the two cells in **E **positive for PPT1 with and without CLN5-TD), whereas overexpression of the ER resident CLN6 did not have an influence on PPT1 trafficking (**G-I**). The CLN5-TD partially affected also the lysosomal targeting of the wild type CLN3 (**J-L**). Scale bar 10 μm.

**Figure 3:**
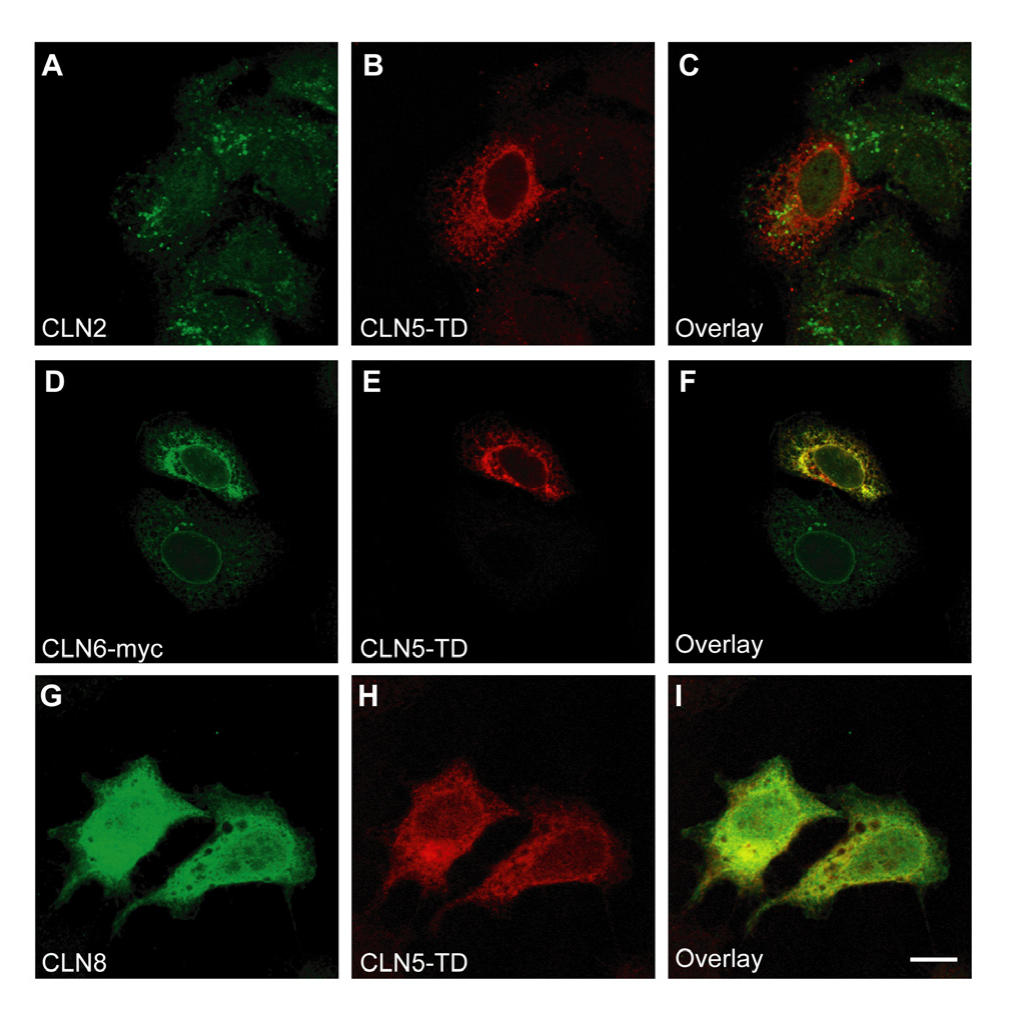
**Consequences of the ER-resident trafficking deficient CLN5 on the localization of CLN2, CLN6 and CLN8 in HeLa cells**. HeLa cells were transiently transfected alone with the trafficking deficient CLN5 (CLN5-TD) carrying the intramolecular flag-tag after aa 330 (**A-C**) or together with wild type CLN6 (**D-F**) or CLN8 (**G-I**). The cells were fixed with methanol 48 h post transfection, stained and analyzed by confocal microscopy. The CLN5-TD did not have an effect on the lysosomal localization of endogenous CLN2 (**A-C**) or on the overexpressed ER resident NCL proteins CLN6 (**D-F**) and CLN8 (**G-I**). Scale bar 10 μm.

To assess whether severe defects in the transport of CLN5 could affect the localization of any of the interacting proteins, co-expressions were performed using a trafficking-deficient hCLN5 polypeptide (CLN5-TD), representing a protein restricted to the ER. The intramolecular insertion of a flag-tag (CLN5-flag330) restrained the CLN5-TD polypeptide into the ER in HeLa cells (Fig. 2A-C). Interestingly, when the wild type PPT1/CLN1 was co-expressed with this ER-resident construct, it was also retained in the ER (Fig. 2D-F). To control the specificity of the observed restriction on PPT1 transport by CLN5, PPT1/CLN1 was also overexpressed together with trafficking-deficient CLN3-flag434 (data not shown) and ER-resident CLN6 (Fig. 2G-I). Neither of these proteins affected the lysosomal trafficking of PPT1/CLN1. The transport of CLN3 from the ER was also delayed when it was co-expressed with the ER-resident CLN5-TD; CLN3 was detected in the lysosomes, but was additionally seen in the ER (Fig. 2J-L). However, the effect was milder compared to that seen with PPT1/CLN1 and therefore was thought to result from problems in protein folding in the ER. Trafficking of the endogenous CLN2 was not altered due to overexpression of CLN5-TD and it was correctly transported to the lysosomes (Fig. 3A-C). As assumed, the ER-resident proteins CLN6 (Fig. 3D-F) and CLN8 (Fig. 3G-I) remained in the ER and were not detectably influenced by the transport defect of CLN5-TD.

### Overexpression of wild type PPT1/CLN1 rescues the lysosomal trafficking of CLN5_FinMajor _polypeptide

**Figure 4:**
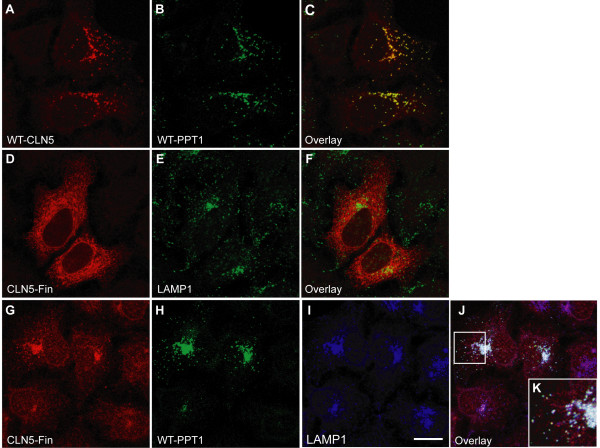
**Consequence of the CLN5_FinMajor _disease mutation on the trafficking of PPT1/CLN1 in HeLa cells**. HeLa cells were transiently transfected with the wt CLN5 together with wt PPT1 (**A-C**), or CLN5_FinMajor _alone (**D-F**), or CLN5_FinMajor _carrying the most common vLINCL_Fin _mutation together with wt PPT1 (**G-K**). The cells were fixed with methanol 48 h post transfection, stained and analyzed by confocal microscopy. The wt proteins co-localized with each other completely (**A-C**). When CLN5_FinMajor _was expressed alone, it was retained in the ER and did not co-localize with the lysosomal marker LAMP-1 (**D-F**). When CLN5_FinMajor _was co-expressed with wt PPT1, the proteins co-localized in the lysosomes (**G-K**). Scale bar 10 μm.

As the most dramatic influence on the localization of the binding partners of CLN5 was seen for PPT1/CLN1, it was of interest to study if the expression of CLN5 possessing the most common vLINCL_Fin _disease-causing mutation (p. Y392X, CLN5_FinMajor_) would also influence the trafficking of PPT1/CLN1. The CLN5_FinMajor _mutation results in a truncated polypeptide lacking 16 amino acids from the C-terminus [17] and it has been demonstrated to localize to the ER and Golgi instead of lysosomes [18]. Importantly, this mutant protein represents an ER exit-competent CLN5, in contrast to the CLN5-TD which is a strict ER-resident. We first expressed the wild type PPT1 with wild type CLN5 in HeLa cells and found that the two proteins completely co-localized in the lysosomes (Fig. 4A-C). The mutated CLN5_FinMajor _polypeptides expressed alone did not show co-localization with the lysosomal marker, LAMP-1, but were retained in the ER in HeLa cells (Fig. 4D-F). Interestingly, simultaneous overexpression of wild type PPT1 with CLN5_FinMajor _resulted in a positive effect on the trafficking of the mutated CLN5, and both proteins were found in the lysosomes (Fig. 4G-K and Additional file 2). To verify the results obtained in HeLa cells, we repeated the experiments also in human neuroblastoma cells (SH-SY5Y). Simultaneous overexpression of wild type PPT1/CLN1 and CLN5_FinMajor _in SH-SY5Y cells again resulted in co-localization of the two proteins in LAMP-1 positive late endosomes/lysosomes, whereas the CLN5_FinMajor _expressed alone was detected only in the ER (Additional file 3). Wild type PPT1/CLN1 seemed thus to have a positive effect on the transport of CLN5_FinMajor _when overexpressed in the same cell, being able to facilitate the trafficking of the mutated CLN5 from the ER into the late endosomal compartments.

### PPT1/CLN1 mutants retained in the ER do not restrict the lysosomal trafficking of CLN5

**Figure 5:**
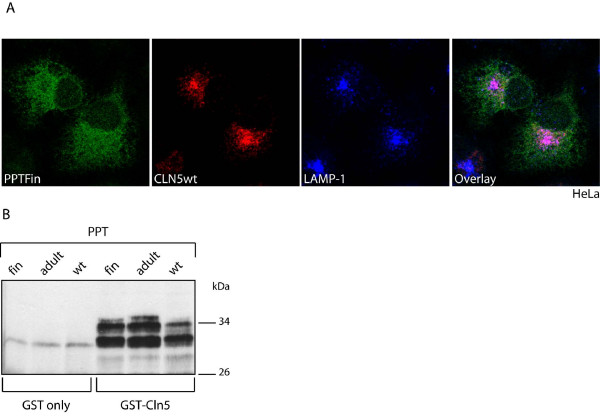
**Consequences of the INCL disease mutations on the trafficking of CLN5**. **(A) **HeLa cells were transiently transfected with wt CLN5 and PPT1_Fin_, carrying the most common INCL causing mutation. Cells were fixed with methanol 48 h post transfection, stained and analyzed by confocal microscopy. When wt CLN5 was co-expressed with wt PPT1_Fin_, wt CLN5 was able to traffic to lysosomes, whereas PPT1_Fin _retained in the ER. Scale bar 10 μm. **(B) **The GST-mCLN5 was used to pull down PPT1 carrying INCL disease mutations (PPT_Fin _or the adult-onset causing mutation) and wild type CLN1/PPT1 from COS-1 cell lysates. Both PPT_Fin _and the adult-onset mutations maintained their interaction with CLN5.

As PPT1/CLN1 was found to facilitate trafficking of the mutated CLN5_FinMajor_, we wanted to study whether it is a prerequisite for the lysosomal trafficking of CLN5. We overexpressed PPT1/CLN1 carrying either the most common INCL disease mutation (PPT1_Fin_, p. R122W) [34] or the adult-onset causing mutation (PPT1_Adult_, p. G108R) [35] together with the wt CLN5 in HeLa cells. The localization of the proteins was then studied by confocal microscopy. Both PPT1_Fin _(Fig. 5A) and PPT1_Adult _(data not shown, [26]) localized to the ER, whereas the wt CLN5 was found in the lysosomes (Fig. 5A). The mutated PPT1/CLN1 proteins were never detected in lysosomes suggesting that CLN5 lacks the positive targeting effect found in the opposite situation with wt PPT1/CLN1. This could, however, result from a defective interaction between the two proteins due to PPT1 mutations. To study this, we used GST-mCLN5 to pull down overexpressed PPT1_Fin _and PPT1_Adult _proteins from the COS-1 cell lysates. Both of the mutated PPT1/CLN1 proteins were pulled down with CLN5, similarly to the wt protein (Fig. 5B), demonstrating that the interaction between the two proteins was maintained. These data indicated that although PPT1/CLN1 can facilitate the lysosomal trafficking of CLN5_FinMajor_, CLN5 cannot rescue the restricted trafficking of the mutated PPT1/CLN1 to the lysosomes.

### Co-immunoprecipitation and compensatory mRNA expression support the relationship between CLN1 and CLN5

**Figure 6:**
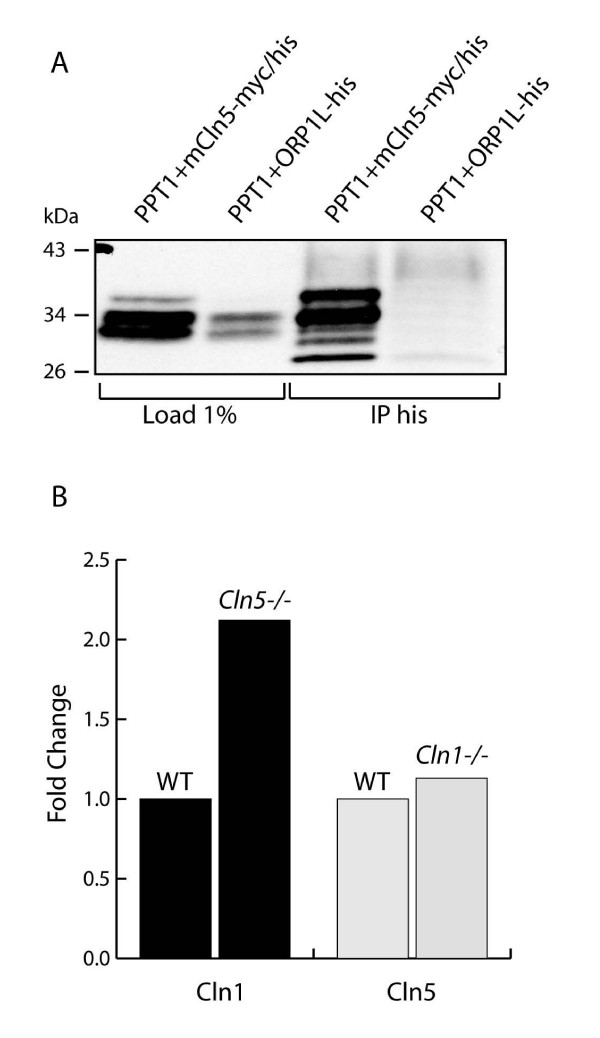
**Association between CLN5 and PPT1/CLN1**. **(A) **COS-1 cells were transiently transfected with PPT1/CLN1 together with mCLN5-myc/his or ORP1L-his. Co-immunoprecipitation was performed with a his-specific antibody, followed by Western blot detection for PPT1/CLN1. CLN5 was found to bind specifically with PPT1/CLN1, confirming the interaction between the proteins. **(B) **RNA was extracted from the whole cortical area of four *Cln5*^-/- ^mice, four *Cln1*^-/- ^mice and four of their respective wt littermates and pooled together into two wild-type and two knock-out samples. Real time RT-PCR was then performed to measure expression of the two NCL proteins. The value for the wt gene expression was set to 1 and *Cln5 *and *Ppt1 *expression values are shown relative to that. Expression of CLN1 (black columns) was over two-fold up-regulated in the 4-month-old *Cln5*^-/- ^mice compared to that of the wt. The expression of CLN5 (gray columns) was only slightly altered in *Cln1*^-/- ^mice of the same age.

Both our pull-down and trafficking experiments indicated a strong interaction between CLN1 and CLN5, but since this interaction was not detected in the previous co-immunoprecipitation assay [23], we wanted to strengthen our data with additional experiments. Therefore, we also performed a co-immunoprecipation assay and analyzed the mRNA expression of the binding partner protein both in *Cln1 *and *Cln5 *deficient mice. In the co-immunoprecipitation assay, PPT1/CLN1 was expressed together with mCLN5-myc/his or with a control protein ORP1L-his [32] in COS-1 cells. mCLN5-myc/his and ORP1L-his were immunoprecipitated with a his-specific antibody followed by PPT1/CLN1 detection by Western blot analysis. PPT1/CLN1 co-immunoprecipitated with CLN5, especially the tri- and diglycosylated forms, supporting the binding of the premature ER forms of PPT1/CLN1 [26] to CLN5. The control protein ORP1L showed no binding to PPT1/CLN1 (Fig. 6A).

Real time PCR was performed from the cortices of wt, *Cln1 *and *Cln5 *deficient mice to detect the possible dependence of the disruption of one binding partner on the expression of the other. Analyses of mRNA expression levels in brain tissues showed significant upregulation of the *Cln1 *mRNA in the four-month old *Cln5 *deficient mice, indicating a possible compensatory response. The mRNA levels of *Cln5 *were only slightly upregulated in the brain tissue of the *Cln1 *deficient mice (Fig. 6B). Altogether, these results strengthened our novel findings and suggested a possible functional connection between CLN1 and CLN5 proteins.

### Interaction of CLN5 with the subunits of F_1_-ATP synthase

**Figure 7:**
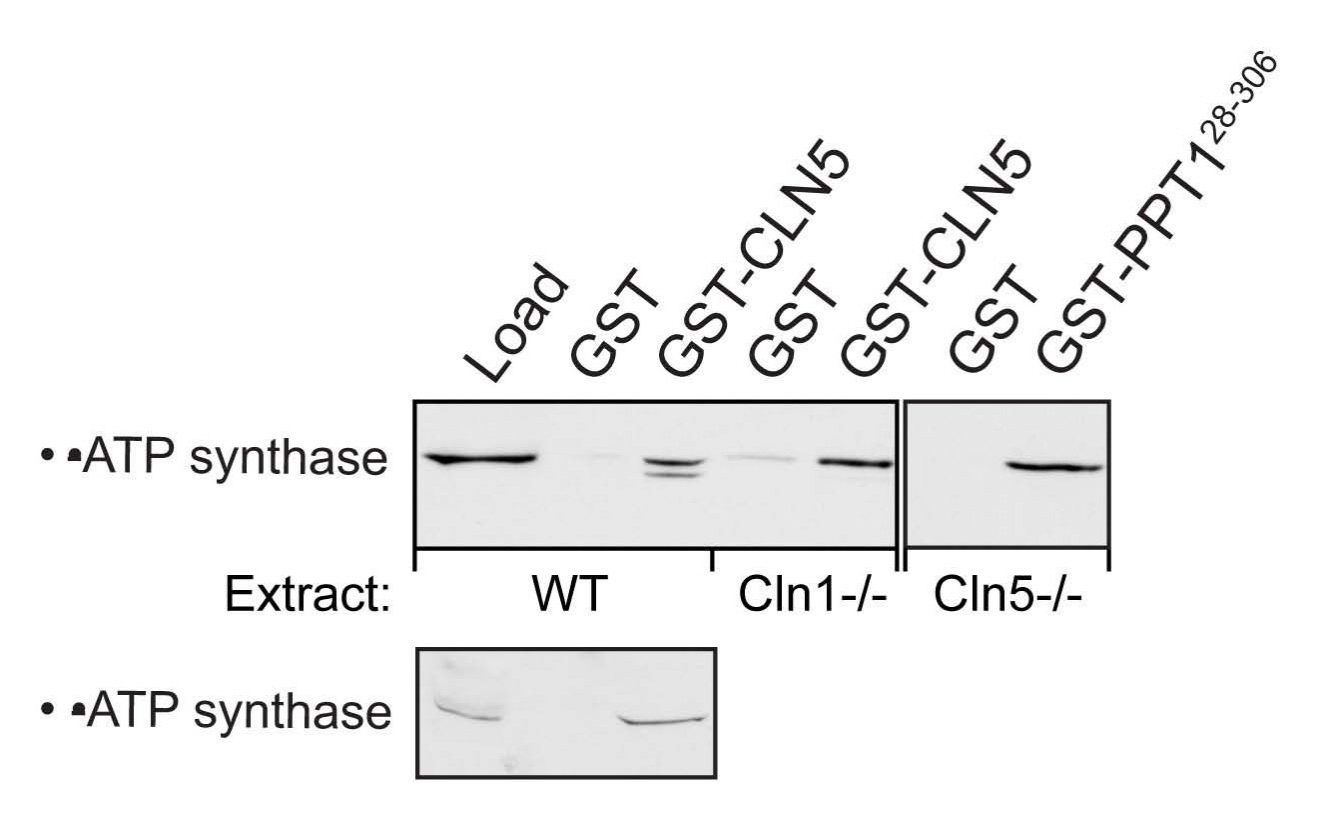
**Interaction of CLN5 with the α- and β-subunits of the ATP synthase**. GST-hPPT1 and GST-mCLN5 fusion proteins were used to pull down interacting proteins from enriched lysosomal/mitochondrial fraction extracted from the liver of wild type *Cln1*^-/- ^and *Cln5 *^-/- ^mice. The bound proteins were analyzed by Western blot using monoclonal antibodies for α- and β-ATP synthase. Load = 10 μg of total protein from mouse liver extract.

We have recently shown that PPT1/CLN1 co-purifies in a large protein complex and interacts with the F_1_-complex of ATP synthase [26,31]. Due to our novel findings, we tested whether CLN5 would also interact with the F_1_-complex. We used GST-mCLN5 to pull-down proteins from an enriched lysosomal/mitochondrial fraction prepared from mouse liver. Interestingly, both α- and β-subunits of the F_1_-complex showed interaction with CLN5 (Fig. 7). To test whether PPT1 or CLN5 require each other for their respective interactions with the F_1_-complex, the experiment was repeatedly performed using lysosomal/mitochondrial fractions extracted from both *Cln1*^-/- ^and *Cln5*^-/- ^mice lacking the respective proteins. The β-subunit was able to bind both GST-hPPT1 in the absence of CLN5 and GST-mCLN5 in the absence of PPT1, indicating that the proteins interact with the β-subunit independent of each other (Fig. 7). These results show that in addition to an interaction between PPT1/CLN1 and CLN5, the proteins share another binding partner not belonging to the group of NCL proteins.

## Discussion

Clinical and neuropathological similarities in NCL disorders may result from functional redundancy or co-operation of different NCL proteins. Initial evidence of co-operation has previously been obtained from activity measurements and gene expression analyses. For example, CLN2/TPP1 activity has been shown to be elevated in other forms of NCL [23,36,37]. Furthermore, the mRNA expression levels of a variety of NCL genes have been reported to be altered in different NCLs [22]. Recent studies of animal models have further supported a common mechanism in the disease pathogenesis of the NCLs. Selective loss of interneurons, early defects on thalamocortical neuron survival as well as early glial responses are detected in different mouse models as well as in large animal models [8,38]. Molecular interactions between NCL proteins have also been reported previously [23,39]. Here we continued the search for NCL protein interactions and their intracellular consequences. We demonstrate that CLN5 has molecular connections to at least to five other NCL proteins, namely CLN1/PPT1, CLN2/TPP1, CLN3, CLN6 and CLN8, suggesting a central role for CLN5 in the NCL network.

Mutations of CLN5 and consequent trafficking defects can result in functional consequences on the interacting proteins, as well as in changes in their distribution. Most NCL proteins are not, however, detectable endogenously by immunofluorescence analyses using the currently available antibodies and therefore this hypothesis could not be tested in patient fibroblasts. However, simultaneous overexpression of binding partners with the modified, transport incompetent CLN5-TD (CLN5-flag330) construct suggested that the interaction between PPT1/CLN1 and CLN5 is strong and occurs already in the ER, where the interactions between CLN5 and the two ER resident NCL proteins, CLN6 and CLN8, would also naturally occur. Since CLN5 resides in the lumen of intracellular organelles, interaction between CLN5 and the transmembrane proteins must be mediated by the lumenal domains of these proteins. The finding that NCL interactions can occur already in the ER is important since previous studies have also suggested that the ER is an important organelle for the function and/or trafficking of NCL proteins. For example, CLN3 carrying the most common JNCL mutation [40] has been reported to preserve a significant function in the ER. Although the mutated CLN3 is retained in the ER, it was shown to be able to affect the size of lysosomes [41]. Furthermore, our data suggest that TPP1/CLN2, another lysosomal enzyme, is likely to interact with CLN5 only in the late endosomes/lysosomes. This was supported both by the interaction analysis and the unaffected transport of CLN2. Therefore, the present data suggest that interactions between CLN5 and other NCL proteins can occur along the secretory pathway, and the interactions are not strictly dependent on the steady-state localization or the solubility of the NCL proteins.

Based on our trafficking experiments, the main focus was on the interaction between PPT1/CLN1 and CLN5. Unlike the transport deficient, strictly ER-resident CLN5-TD, the ER exit competent CLN5_FinMajor _did not prevent the lysosomal trafficking of PPT1 but rather, PPT1 was able to facilitate the trafficking of the mutated CLN5 from the ER and Golgi to the lysosomes. Lysosomal proteins have also previously been shown to assist each other in their lysosomal trafficking. For example, the CLC7 chloride transporter, involved in an NCL-like disorder in mouse, has been shown to facilitate the transport of Ostm1 to the lysosomes, where the proteins act together in lysosomal chloride transport [42]. It was also recently demonstrated that the lysosomal membrane protein LIMP-2 is required for the mannose 6-phosphate receptor-independent targeting of β-glucocerebrosidase. Interestingly, the overexpression of LIMP-2 was also able to facilitate the transport of the mutated, trafficking deficient β-glucocerebrosidase from the ER to the lysosomes [43]. Both PPT1 and CLN5 are soluble intravesicular proteins and are not able to interact with cytoplasmic sorting and transport machinery per se, like CLC7 and LIMP-2. However, trafficking of CLN1/PPT1 has been reported to show properties different from classic lysosomal enzymes [26] and we have recently discovered that also CLN5 can use M6PR-independent pathways for its lysosomal trafficking (our unpublished observations). Therefore, it is possible that the formation of the CLN1-CLN5 complex may be important for utilizing other trafficking pathways than the classical M6PR pathway. In which circumstances this is required in vivo, remains to be studied in further experiments.

A close connection between PPT1/CLN1 and CLN5 has already been suggested in previous studies. The proteins share similar expression patterns in the mouse brain and in the prenatal human brain [16,21,44,45]. Our recent global gene expression profiling analyses of the *Cln1*^-/- ^and *Cln5*^-/- ^mouse brains implicated a common defective pathway mediated by phosphorylation and potentially affecting the maturation of axons and neuronal growth cones [46]. Here, we provide further evidence for a tight relationship between the two proteins by showing not only the interaction between the proteins but also, demonstrating significantly increased expression levels of *Cln1 *mRNA in the *Cln5*^-/- ^mouse brain tissue. This suggests a possible compensatory role for PPT1 in CLN5 deficiency. Functional connection of CLN5 and PPT1 is also suggested by the shared interaction partner not belonging to the NCL protein family, the F_1_-ATP synthase. The ectopic F_1_-ATP synthase has been shown to function as an apoA-I receptor on the plasma membrane [47] and both the amount of the F_1_-complex as well as the uptake of apoA-I have been shown to be increased in *Cln1*^-/- ^mouse neurons [31]. Therefore, both PPT1 and CLN5 could be connected to the maintenance of lipid homeostasis. In general, accumulating evidence has indicated dysregulated lipid metabolism in different forms of NCLs and several NCL proteins have been functionally linked to lipid metabolism [[48,49], reviewed in [50-52]].

## Conclusion

In this study, we show novel interactions between the neuronal ceroid lipofuscinosis protein CLN5 and five other NCL proteins. Consequently, our study strengthens the long-term hypothesis of a common cellular pathway behind the NCLs and suggests that different mutations in a given NCL protein may lead to different pathological outcomes through variable distinct effects on the NCL protein network. The strongest interaction was detected between CLN5 and PPT1/CLN1, and PPT1/CLN1 was shown to be able to contribute to the intracellular trafficking of the mutated CLN5, the phenomenon, which may be important when planning the therapy for vLINCL_Fin_. Deficiency of *Cln5 *was also shown to result in upregulation of *Cln1 *expression in the mouse brain, suggesting a dependency for CLN1/PPT1 over CLN5. For the first time, the two NCL proteins were shown to share an interaction partner outside the NCL protein spectrum, since CLN5 and PPT1 both interacted with the F_1_-complex of the ATP synthase. This finding may be important in characterization of the cellular functions of the NCL proteins.

## Authors' contributions

AL carried out the immunofluorescent analyses, participated in the design of the study and drafted the manuscript. CvS participated in the performance of the immunofluorescent analyses, the study design and drafting of the manuscript. CvS also carried out the mRNA expression analyses. CH carried out most of the GST pull-down experiments together with MLS and TS. AJ and AK conceived of the study, participated in its design and coordination and helped to draft the manuscript. All authors read and approved the final manuscript.

## Supplementary Material

Additional file 1 **Interaction of CLN5 with endogenous NCL proteins in HeLa cells**. The mouse *Cln5*-cDNA was expressed as a GST fusion protein and used for pull down analyses of endogenous NCL proteins from HeLa cell lysates. The bound proteins were immunoblotted and detected with specific antibodies: CLN2, rabbit polyclonal antibody 7951 [23], CLN3, polyclonal 385 antibody [21] PPT1, rabbit polyclonal antibody 8414 [19], and with anti-CLN6 antibody, which was a generous gift from Dr. S. Mole (London, UK) (Wheeler et al. Am J Hum Genet 70, 2002). Click here for file

Additional file 2 **Co-expression of the mutated CLN5 and wtPPT1 in HeLa cells**. HeLa cells were transiently transfected with the mutated CLN5Fin (A) and wt CLN1/PPT1 (B). The cells were fixed with methanol 48 h post transfection, stained and analyzed by confocal microscopy. The Golgi complex is stained with GM130 (C). CLN5Fin and wt PPT co-localized only partially with the Golgi complex (D-F). Scale bar 10 μm. Click here for file

Additional file 3 **Facilitated lysosomal trafficking of the mutated CLN5 by PPT1 overexpression in SH-SY5Y cells**. Human neuroblastoma cells (SH-SY5Y), were transiently transfected with wt CLN1/PPT1 and CLN5-Fin, carrying the most common vLINCL(Fin) causing mutation (A-E), or with CLN5-Fin alone (F-H). The cells were fixed with methanol 48 h post transfection, stained and analyzed by confocal microscopy. When CLN5-Fin was co-expressed with wt PPT1, CLN5-Fin was able to traffic to lysosomes with PPT1 (A-E). When CLN5-Fin was expressed alone, it retained in the ER (F-H). Scale bar 10 μm. Click here for file
