# Supplementary material for: Novel interactions of CLN5 support molecular networking between Neuronal Ceroid Lipofuscinosis proteins
Source: BMC Cell Biol. 2009 Nov 26;10:83. doi: 10.1186/1471-2121-10-83 (PMC2790443; doi:10.1186/1471-2121-10-83)
Supplement: Additional file 1 — Interaction of CLN5 with endogenous NCL proteins in HeLa cells. The mouse Cln5-cDNA was expressed as a GST fusion protein and used for pull down analyses of endogenous NCL proteins from HeLa cell lysates. The bound proteins were immunoblotted and detected with specific antibodies: CLN2, rabbit polyclonal antibody 7951 [23], CLN3, polyclonal 385 antibody [21] PPT1, rabbit polyclonal antibody 8414 [19], and with anti-CLN6 antibody, which was a generous gift from Dr. S. Mole (London, UK) (Wheeler et al. Am J Hum Genet 70, 2002). [file 1471-2121-10-83-S1.PDF]

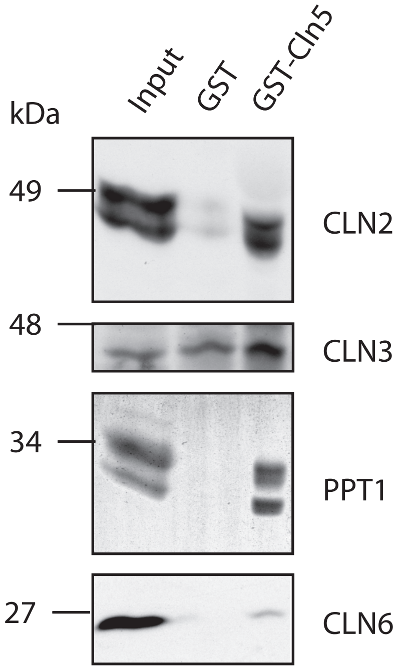


**Additional file 1.**

**Interaction of CLN5 with endogenous NCL proteins in HeLa cells.**
The mouse Cln5-cDNA was expressed as a GST fusion protein and used for pull-down analyses of endogenous NCL proteins from HeLa cell lysates. The bound proteins were immunoblotted and detected with specific antibodies: CLN2, rabbit polyclonal antibody 7951 [23], CLN3, polyclonal 385 antibody [21] PPT1, rabbit polyclonal antibody 8414 [19], and with anti-CLN6 antibody, which was a generous gift from Dr. S. Mole (London, UK) (Wheeler et al. Am J Hum Genet 70, 2002).
