# Supplementary material for: Novel interactions of CLN5 support molecular networking between Neuronal Ceroid Lipofuscinosis proteins
Source: BMC Cell Biol. 2009 Nov 26;10:83. doi: 10.1186/1471-2121-10-83 (PMC2790443; doi:10.1186/1471-2121-10-83)
Supplement: Additional file 2 — Co-expression of the mutated CLN5 and wtPPT1 in HeLa cells. HeLa cells were transiently transfected with the mutated CLN5Fin (A) and wt CLN1/PPT1 (B). The cells were fixed with methanol 48 h post transfection, stained and analyzed by confocal microscopy. The Golgi complex is stained with GM130 (C). CLN5Fin and wt PPT co-localized only partially with the Golgi complex (D-F). Scale bar 10 μm. [file 1471-2121-10-83-S2.PDF]

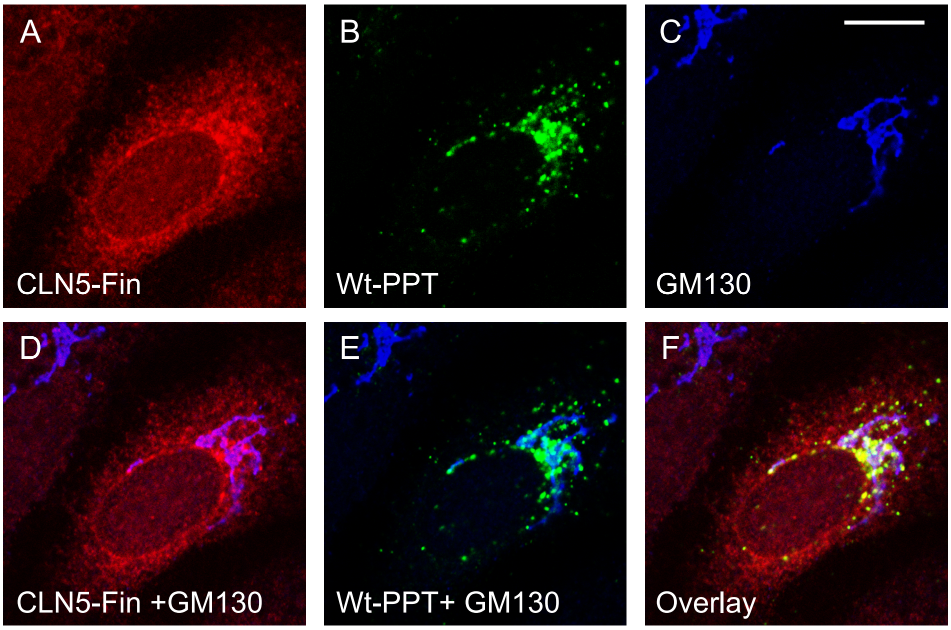

Additional file 2.

Co-expression of the mutated CLN5 and wt PPT1 in HeLa cells
HeLa cells were transiently transfected with the mutated $CLN5_{Fin}$ (A) and wt CLN1/PPT1 (B). The cells were fixed with methanol 48 h post transfection, stained and analyzed by confocal microscopy. The Golgi complex is stained with GM130 (C). $CLN5_{Fin}$ and wt PPT co-localized only partially with the Golgi complex (D-F). Scale bar 10 μm.
