# Supplementary material for: Novel interactions of CLN5 support molecular networking between Neuronal Ceroid Lipofuscinosis proteins
Source: BMC Cell Biol. 2009 Nov 26;10:83. doi: 10.1186/1471-2121-10-83 (PMC2790443; doi:10.1186/1471-2121-10-83)
Supplement: Additional file 3 — Facilitated lysosomal trafficking of the mutated CLN5 by PPT1 overexpression in SH-SY5Y cells. Human neuroblastoma cells (SH-SY5Y), were transiently transfected with wt CLN1/PPT1 and CLN5-Fin, carrying the most common vLINCL(Fin) causing mutation (A-E), or with CLN5-Fin alone (F-H). The cells were fixed with methanol 48 h post transfection, stained and analyzed by confocal microscopy. When CLN5-Fin was co-expressed with wt PPT1, CLN5-Fin was able to traffic to lysosomes with PPT1 (A-E). When CLN5-Fin was expressed alone, it retained in the ER (F-H). Scale bar 10 μm. [file 1471-2121-10-83-S3.PDF]

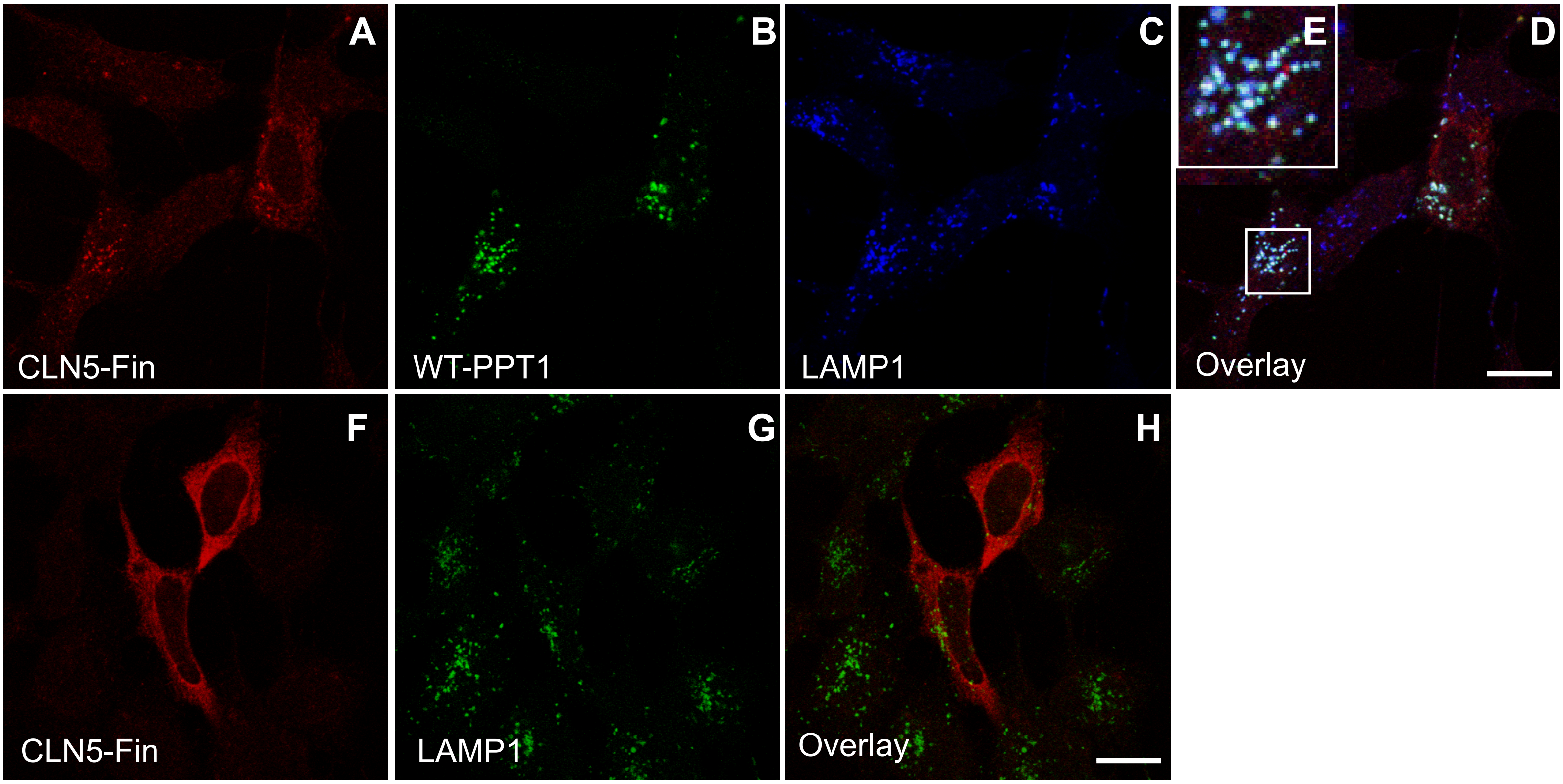

Additional file 3.

Facilitated lysosomal trafficking of the mutated CLN5 by PPT1 overexpression in SH-SY5Y cells
Human neuroblastoma cells (SH-SY5Y), were transiently transfected with wt CLN1/PPT1 and CLN5-Fin, carrying the most common vLINCL(Fin) causing mutation (A-E), or with CLN5-Fin alone (F-H). The cells were fixed with methanol 48 h post transfection, stained and analyzed by confocal microscopy. When CLN5-Fin was co-expressed with wt PPT1, CLN5-Fin was able to traffic to lysosomes with PPT1 (A-E). When CLN5-Fin was expressed alone, it retained in the ER (F-H). Scale bar 10 μm.
